# Efficacy of coping mechanisms used during COVID-19 as reported by parents of children with autism

**DOI:** 10.1371/journal.pone.0283494

**Published:** 2023-04-13

**Authors:** Florence Wang, Idil Memis, Jennifer S. Durocher, Emily Furar, Leylane Cavalcante, Rebecca S. Eshraghi, Andrea C. Samson, Jo Van Herwegen, Daniel Dukes, Michael Alessandri, Rahul Mittal, Adrien A. Eshraghi

**Affiliations:** 1 Hearing Research and Communication Disorders Laboratory, Department of Otolaryngology, Miller School of Medicine, University of Miami, Miami, Florida, United States of America; 2 Department of Psychology, University of Miami, Coral Gables, Florida, United States of America; 3 Institute of Special Education, University of Fribourg, Fribourg, Switzerland; 4 Faculty of Psychology, Unidistance Suisse, Brig, Switzerland; 5 Department of Psychology and Human Development, UCL, Institute of Education, University College London, London, United Kingdom; 6 Swiss Center for Affective Sciences, University of Geneva, Geneva, Switzerland; 7 Department of Neurological Surgery, Miller School of Medicine, University of Miami, Miami, Florida, United States of America; 8 Department of Pediatrics, Miller School of Medicine, University of Miami, Miami, Florida, United States of America; Radboud University: Radboud Universiteit, NETHERLANDS

## Abstract

The COVID-19 pandemic’s alterations to daily life have been especially challenging for families with Autism Spectrum Disorder (ASD), worsening the core features of ASD and overall mental health. With the increased need for effective coping, the current retrospective study used data from a survey regarding parent reports of how often their child with ASD used certain coping strategies (frequency), as well as the extent to which they felt their child benefitted from their use (efficacy) in mitigating stress during the pandemic. This retrospective study Repeated measures ANOVAs were conducted to evaluate whether there were significant differences in both frequency and efficacy ratings for each coping strategy, for the entire sample as well as for three children’s age groups. Using Spearman’s rank-order correlations, correlation coefficients between the frequency and efficacy of each coping strategy were explored. Results revealed that maladaptive strategies were used more frequently than adaptive strategies, while parent routine as the most frequently used and efficacious for all age groups. Additionally, for adaptive strategies, humor and focusing on the positive had the strongest correlations between frequency and efficacy ratings amongst all age groups. Of the maladaptive strategies, repetitive behaviors, rumination, and isolation had the strongest correlations for the youngest, middle, and oldest age groups, respectively. Further, for each age group, the adaptive coping strategies had stronger correlations between frequency and efficacy than the maladaptive ones. It is our hope that the results of this study will lay the foundation for developing adaptive coping strategies to alleviate stress in children with ASD. Further investigations using a larger cohort are warranted to determine effective coping strategies for individuals with ASD across a range of situations, including acute stressors (such as future public health emergencies and natural disasters), as well as common daily stressors.

## Introduction

Autism Spectrum Disorder (ASD) is a neurodevelopmental disorder which presents with deficits in social communication as well as specific and restricted patterns of behavior and interests [1–3]. The prevalence of ASD in children within the United States is currently 1 in 44 [4]. In addition to the core symptoms of ASD, many individuals with ASD experience various co-occurring conditions. The most common of these co-occurring diagnoses include attention-deficit/hyperactivity disorder (ADHD), anxiety, depression, intellectual disability, and epilepsy [5]. In order to manage symptoms and to improve the overall wellbeing, developmental outcomes and quality of life of individuals with ASD, interventions and services are often utilized [6]. Service use commonly includes a combination of applied behavior analysis (ABA) therapy, speech and occupational therapies, parent-mediated interventions, and school-based services as well as specialized education [5, 7]. As individuals with ASD follow a rigid routine, events like health crisis that involve swift implementation of mandates, which disrupt familiar daily routines can be especially stress-inducing for this population.

On March 11, 2020, the World Health Organization (WHO) declared the coronavirus disease 2019 (COVID-19) a pandemic and, rapidly, state mandates regarding stay-at-home orders were implemented, instituting a nationwide shutdown [8]. The urgency of the situation and the abrupt changes which followed left many families of children with ASD in a state of uncertainty due to the alterations to the typical environments and routines their children had become accustomed to. These disruptions of essential services and therapies have been hypothesized to result in worsening autism symptoms, increased behavioral challenges and decreased mental well-being for children with ASD [9, 10]. A clear understanding of the impact of the COVID-19 pandemic on the ASD population, is vital to allow for better preparation for future public health emergencies and pandemics in order to reduce the adverse effects for these individuals.

Studies performed regarding the possible emotional and behavioral effects of COVID-19 on children who were diagnosed with ASD commonly reported increases in anxiety and worries, particularly regarding social isolation, physical distancing, and school [9, 11–14]. According to researchers who surveyed parents of children with ASD, the stress brought on by the pandemic has also been associated with increases in behavioral problems, and an exacerbation of core ASD symptoms, in terms of both frequency and intensity [9, 15–17]. Alterations in the ways in which services are received by children with ASD, as well as shifting family dynamics due to stay-at-home orders, have resulted in an increased risk of negative symptomatology within this cohort. This negative symptomatology could include maladaptive behaviors such as aggression or acts of self-harm, “skill loss or stagnation,” and repetitive behaviors, which are considered to be likely stressors for the child with ASD as well as the family [9, 16–21]. The notably high levels of distress experienced by both the family and the child with ASD during these unexpected times demand a determination of effective mechanisms for these individuals to increase coping and decrease stress [9, 10].

Emotion regulation and coping strategies, however, are understudied within the ASD community, especially regarding their use and efficacy in children with ASD [22, 23]. Coping and emotion regulation are considered to be distinct but overlapping constructs, with convergence and divergence in their definitions, measurement and treatment [24]. One of the hallmark distinctions between these constructs is that coping specifically involves strategies employed in response to stressful situations [24–26], whereas emotion regulation is utilized under both stressful and non-stressful situations and aims to change the intensity of both negative and positive emotions [24, 25]. In this respect, then, “coping is a subset of emotion regulation enacted in response to stressful events or circumstances” [24].

Both emotion regulation (ER) and coping strategies are important as they assist in the ability to adjust to new situations, changes in the environment, or novel stimuli [23, 27]. These strategies can also be categorized as adaptive or maladaptive [23, 25, 26, 28]. Those which invoke more negative long-term effects are considered to be maladaptive, while those with positive consequences following continued use are labeled as adaptive. In typically developing individuals, increased use of adaptive coping/ER strategies (such as distraction, and cognitive reappraisal and restructuring), are negatively correlated with psychopathology, while responses such as emotional suppression, rumination, denial, and avoidance, which are frequently considered maladaptive, have been linked with poor mental health and psychopathology [22, 25].

The use of adaptive coping/ER strategies in children with ASD appear to be uniquely challenging. Compared to their typically developing counterparts, individuals with ASD appear to be more likely to utilize maladaptive strategies such as expressive suppression (e.g., holding emotions inside), rumination, avoidance and venting [22, 29] and less likely to use adaptive strategies such as seeking social support or cognitive reappraisal [23, 29, 30]. While traditionally considered to be maladaptive behaviors rather than coping strategies, an additional line of research has conceptualized commonly observed problematic behaviors in ASD as attempts to “cope” with their environment [31]. In this model, maladaptive behaviors such as social avoidance, restricted and repetitive behaviors and even self-injurious behaviors may serve help as to help reduce feelings of negative affect and anxiety [31, 32]. Specifically, repetitive behavior may serve a self-regulatory (and possibly adaptive) function which serves to prevent individuals from becoming overwhelmed by allowing them to deal with sensory overload and/or move their attention away from what is distressing them [33–35]. Repetitive behaviors may also help reduce anxiety for individuals with ASD by “reintroducing certainty, control or consistency” to anxiety producing situations [36]. Likewise, aggression (although clearly a maladaptive behavior and strategy) may be used by individuals with ASD in response to stress or anger [37] in order to “communicate distress” around the “uncertainties of the pandemic” [38]. Further, Chin and colleagues described “acting out “(e.g., aggression) as a “disengagement coping strategy” used by children with autism in their study, which “reduced or managed stress associated with stressors that could not be controlled” [39]. In this regard, aggression and repetitive behavior may reflect maladaptive coping strategies which might be employed to cope with stress brought about due to the pandemic.

The primary objective of the current study was to evaluate the frequency and efficacy of various adaptive as well as maladaptive coping strategies employed by children with ASD during the COVID-19 pandemic in three different age groups (2–9 years old, 10–15 years old, and 16–21 years). In addition, we were also interested in the relationship between the frequency of 14 specific coping strategies and their perceived efficacy, as “just because a strategy is deployed more frequently does not necessarily mean it is more effective” [40]. However, relatively little research has examined the frequency and effectiveness of coping strategy use together, and no studies to date have examined this within a sample of individuals with ASD to our knowledge. In a study of coping strategy use in patients with chronic pain, Roditi and colleagues [41] grouped participants into four groups based on their frequency and effectiveness ratings, finding the following proportions in their sample: frequent use of effective strategies (48.3%), infrequent use of ineffective strategies (30.1%), frequent use of ineffective strategies (19.2%), and infrequent use of effective strategies (1.6%). Therefore, while we might expect frequency and effectiveness ratings to be positively correlated (e.g., frequent and effective; infrequent and ineffective), we may also fail to observe significant correlations (e.g., ineffective regardless of whether frequently or infrequently used; effective regardless of whether frequently or infrequently used). Thus, these correlational analyses were exploratory and meant to serve as a foundation for future examination in subsequent research.

Using national survey data, we determined mean differences in strategy use and effectiveness for the overall sample and for the three age groups, as well as correlation coefficients between frequency and efficacy ratings, for each coping strategy reported. Our goal was to better understand the use and efficacy of various coping strategies among individuals with ASD, which may have important implications for future public health crisis, as well as for interventions designed to cope with stressors more broadly.

## Methods

We performed a retrospective study comparing different age groups on the frequency of various coping strategies in individuals with ASD during the COVID-19 pandemic and assessed the efficacy of these strategies on reducing stress due to COVID-19, based on a survey provided to parents and caregivers (hereafter referred to as ‘parents’) of children with ASD.

### Participants

The present study focuses on a subsample of a larger international sample which included individuals with special educational needs and disabilities (SEND) from over 50 countries. For the current study, our subsample included only parents of individuals with autism spectrum disorder (ASD) within the U.S. (n = 309). We further restricted our sample to include only school-age children with ASD, ages 3 to 21 (n = 246), who were reported by their parents as being aware of the COVID-19 situation (n = 133; 43.04% of the U.S. sample) (Table 1). Because deficits in abstract thinking among children with ASD can impact their ability to understand COVID-19 related stay at home orders [16, 18], several studies conducted during the pandemic have considered COVID-19 awareness status [12–14, 42, 43]. As our aim was to assess how children with ASD were coping with the pandemic, we included only those children who were aware of the pandemic, as they are more likely to be able to employ the various coping strategies assessed in the survey.

**Table 1:** Study participant demographic characteristics and clinical information (*N* = 133).

	*n*	*%*
**Sex**		
Male	117	87.97
Female	16	12.03
**Age (years)**		
3–9	39	29.32
10–15	58	46.61
16–21	36	27.07
**Intellectual disabilities**		
None	66	49.62
Mild to moderate	60	45.11
Severe	7	5.26
**ASD on medication**	69	51.90
**Other health issues** *	56	42.42
Epilepsy	31	23.31
Asthma	5	3.76
Cardiovascular conditions	16	12.03
Diabetes	1	0.75
Overweight	33	24.81
Other	23	17.29
**Other psychological disorders** *	35	26.32
Sleep conditions	10	7.52
ADHD	19	14.29
Anxiety disorder	17	12.78
OCD	2	1.50
Bipolar disorder	2	1.50
Depression	2	1.50
Other	14	10.52

ADHD: attention deficit hyperactivity disorder; OCD: obsessive compulsive disorder

* They are not mutually exclusive.

Participants of the current study included 133 caregivers/parents of individuals with ASD in the U.S., 121 of whom were women (90.98%), aged 29 to 68 years old (mean = 45.21 years old; *SD* = 7.37 years). Of these participants, 108 (81.20%) had a university degree or advanced degree or equivalent. The majority of individuals with ASD included in this study were male (n = 117, 88.0%) and the overall mean age of the individuals with ASD was 12.25 years (*SD* = 4.67 years).

For analysis purposes, divided our population into three age groups with a comparable number of participants in each group: from 3 to 9 years old (n = 39, 29.32%), from 10 to 15 years old (n = 58, 46.61%) and from 16 to 21 years old (n = 36, 27.07%), respectively corresponding with early childhood, late childhood to early adolescence, and late adolescence, which constitute distinct time periods with differing developmental expectations and programmatic needs. The mean age was 6.67 years old in the 3–9-year-old group (*SD* = 1.69 years), 12.29 years old in the 10–15-year-old group (*SD* = 1.67 years), and 18.22 years old in the 16–21-year-old group (*SD* = 1.78 years). Half of our sample (n = 67; 50.4%) were reported to have mild (45.1%) to severe (5.3%) intellectual disabilities. In addition to ASD, most participants also had an additional health or psychological conditions (Table 1). Before COVID-19, 98.5% (n = 131) of the children lived with their parents (versus 1.5% who lived in group home, supported living, or institutional or similar settings) and this proportion remained stable during COVID-19.

### Survey

A survey entitled “COVID-19 Crisis Response Survey for Families of Individuals with Special Needs” was made available in 16 languages through an online platform (https://www.specialneedscovid.org/) and is fully available here: http://osf.io/5nkq9 DOI:10.17605/OSF.IO/5NKQ9. This survey resulted from a multinational collaborative and was completely anonymous. The survey took about 30 minutes to complete, and contained questions regarding mental health, routine alterations, family relations, and changes in access to institutional and therapeutic services at three time points: before the COVID-19 pandemic (prior to March 2020), at the start of the COVID-19 crisis (beginning in March 2020), and at the time of the survey (on or after April 9^th^, 2020). While the international survey was distributed to a broader population of parents of individuals with special educational needs and disabilities (SEND), only those with parent reported ASD diagnosis within the US were included in the current study’s final sample. All collaborators disseminated the link within their own geographical area with data in the US collected between April 9^th^, 2020 and July 1^st^, 2020. This study was approved by the ethics committee of Unidistance Suisse (Nr. 2020-03-00002). The survey was registered on the open science platform (OSF; Van Herwegen, J., Dukes, D., & Samson, A.; 2020, April 9). COVID19 Crisis Response Survey for families of Individuals with Special Needs. Retrieved from http://osf.io/5nkq9 DOI:10.17605/OSF.IO/5NKQ9. Written informed consents were obtained from parents prior to enrollment in the study.

### Data

Parent-reported demographic (i.e., participant age, sex) and clinical data (i.e., intellectual disabilities, co-occurring health conditions, and psychological disorders) were collected. The survey consisted of 14 questions regarding children and parents’ coping strategies used during COVID-19. Twelve children’s strategies were categorized as adaptive or maladaptive in relation to their long-term consequences and in accordance with other research in this area [14], as described in Table 2. *Sharing/talking about COVID-19* and *distraction* may be considered as either adaptive or maladaptive strategies depending on the context, as they may serve as “attentional deployment” or “attention-diverting” strategies [14, 15, 44]. To be consistent with other studies in this are [14, 45], we considered them to be adaptive strategies. Two additional strategies were related to parents or caregivers to regulate their child’s emotion (Table 2). Each question was rated on a scale from 1 to 5, where 1 was “very rarely” used / “not at all” effective and 5 was “very frequently” used / “extremely” effective. For the purposes of the current study, ratings of 3 were interpreted as “somewhat frequently used” / “moderately effective,” while ratings of 4 and 5 were interpreted as “often or very frequently used” / “effective or very effective.”

**Table 2:** Categorization of surveyed coping strategies.

	Strategies as described by parents or caregivers	Strategies labels
**Adaptive strategies**	“In order to feel less stressed, my child changes the way he or she is thinking about the situation.”	*Cognitive reappraisal*
	“In order to feel less stressed, my child focuses on positive aspects/views the situation in a different light.”	*Focusing on the positive*
	“In order to feel less stressed, my child tells jokes and engages in humor.”	*Humor*
	“In order to feel less stressed, my child talks about it as often as possible.”	*Sharing/Talking about COVID-19*
	“In order to feel less stressed, my child distracts him or herself as much as possible.”	*Distraction*
**Maladaptive strategies**	“In order to feel less stressed, my child avoids any information about it.”	*Information Avoidance*
	“In order to feel less stressed, my child gets as much information as possible.”	*Information Search*
	“In order to feel less stressed, my child does not express negative emotions.”	*Expressive suppression*
	“In order to feel less stressed, my child ruminates.”	*Rumination*
	“In order to feel less stressed, my child engages in aggressive behaviors towards others around him/her.”	*Aggressive behaviors*
	“In order to feel less stressed, my child isolates himself/herself in his/her room, or another room of the house.”	*Isolation*
	“In order to feel less stressed, my child engages in repetitive behaviors.”	*Repetitive behaviors*
**Parents’ strategies**	“I try to shield my child from the situation as much as possible.”	*Parent shielding*
	“I try to establish a routine in his/her daily life to lower the experienced stress.”	*Parent routine*

### Analysis

We used IBM SPSS Statistics version 28.0.0 to assess significant differences in the frequency and efficacy of coping strategies by performing repeated measures ANOVAs (3 Age Groups x 14 Coping Strategies). Post-hoc pairwise comparisons were conducted using Bonferroni corrections to compare ratings across the entire sample and across age groups. The correlations between frequency and efficacy of coping strategies for reducing stress were evaluated through performing Spearman’s rank-order correlations. For each analysis, we excluded participants with missing data using pairwise exclusions. The threshold of statistical significance retained was set with a *p* < 0.05. Post-hoc power analyses were conducted using G*Power 3.1 using updated effect size estimates from the current data set. The alpha level used for this analysis was p < .05. The post hoc power analyses for all repeated measures analyses with our current sample size of 104 yielded power (1-β err prob) of 1.00, indicating adequate power (i.e., power * .80) to detect significant differences.

## Results

Overall, results indicate that the majority of coping strategies were used relatively infrequently and were not viewed as particularly effective in managing stress even when used, as the mean ratings of frequency and efficacy for the various strategies rarely exceeded values of “3” (“somewhat frequently used” / “moderately effective”). Descriptively, however, *parent routine* was rated as the strategy that was used most frequently (M = 3.67, SD = 1.29) and rated as most effective (M = 3.37, SD = 1.34) across the entire sample. *Parent shielding* was also relatively frequently used (M = 2.58, SD = 1.41) and was rated as relatively efficacious (M = 2.83, SD = 1.42) in comparison to other strategies.

Among specific children’s use of adaptive coping strategies, *distraction* was rated as the most frequently used (M = 2.79, SD = 1.52) and the most efficacious (M = 2.85, SD = 1.38) of the various strategies. This was followed by the use of *humor* (M = 2.06; SD = 1.23), which was also rated as somewhat efficacious (M = 2.45, SD = 1.48). Regarding maladaptive coping strategies, engaging in *repetitive behaviors* was rated as the most frequently used (M = 3.15, SD = 1.54) and the efficacious (M = 2.77, SD = 1.42), followed by *isolation* which was also rated as relatively frequently used (M = 2.81; SD = 1.55) and effective (M = 2.74, SD = 1.48) (Tables 3 and 4). Indeed, of the six most frequently used strategies, the majority were either maladaptive (*repetitive behaviors*, *isolation*, *rumination)* or parent-mediated (*parent routine*, *parent shielding)*, with only one adaptive strategy (*distraction)* being endorsed.

**Table 3:** Descriptive statistics of the frequency of coping strategies (n = 104).

	Strategy	Mean rating	Standard deviation
**Adaptive strategies**	*Distraction*	2.79	1.52
	*Humor*	2.06	1.23
	*Sharing/Talking about COVID-19*	1.82	1.21
	*Focusing on the positive*	1.74	1.06
	*Cognitive reappraisal*	1.61	0.92
**Maladaptive strategies**	*Repetitive behaviors*	3.15	1.54
	*Isolation*	2.81	1.55
	*Rumination*	2.31	1.41
	*Aggressive behaviors*	2.21	1.43
	*Information Avoidance*	2.02	1.37
	*Information Search*	1.83	1.26
	*Expressive suppression*	1.75	1.16
**Parents’ strategies**	*Parent routine*	3.67	1.29
	*Parent shielding*	2.58	1.41

**Table 4:** Descriptive statistics of the efficacy of coping strategies (n = 104).

	Strategy	Mean rating	Standard deviation
**Adaptive strategies**	*Distraction*	2.85	1.39
	*Humor*	2.45	1.48
	*Sharing/Talking about COVID-19*	2.18	1.41
	*Focusing on the positive*	2.07	1.35
	*Cognitive reappraisal*	1.98	1.27
**Maladaptive strategies**	*Repetitive behaviors*	2.77	1.42
	*Isolation*	2.74	1.48
	*Information Avoidance*	2.54	1.47
	*Information Search*	2.21	1.43
	*Rumination*	2.05	1.21
	*Aggressive behaviors*	1.90	1.40
	*Expressive suppression*	1.86	1.19
**Parents’ strategies**	*Parent routine*	3.37	1.34
	*Parent shielding*	2.83	1.42

In order to better understand the distribution of response patterns for the various coping strategies, the proportion of parents rating each strategy as “frequently or very frequently used” (e.g., ratings of 4 or 5) were examined. *Parent routine* was endorsed as frequently used by nearly 60% of parents compared to 27% for *parent shielding*. For adaptive strategies, only *distraction* was reported as frequently used by a substantial proportion of parents (32%). *Focusing on the positive* and *cognitive reappraisal* were reported to be frequently used by only 6% and 3.5% of parents respectively. For maladaptive strategies, the proportion of parents rating strategies as frequently used included: *repetitive behavior* (45%), *isolation* (38%), *aggression* (23%) and *rumination* (22%).

Similar results were found for the effectiveness of the various strategies. *Parent routine* was rated as “effective or very effective” (e.g., ratings of 4 or 5) by 50%, in comparison to 33% for *parent shielding*. With respect to adaptive strategies, the proportion of parents rating the strategy as effective follows: *distraction* (30%), *humor* (28%), *sharing/talking about COVID-19* (20%). For maladaptive behaviors, the proportion of families indicating that the following strategies were effective included: *isolation* (34%), *repetitive behavior* (30%), *information avoidance* (27.5%) *and aggression* (19%).

### Frequency of coping strategies

Repeated measures ANOVAs with Greenhouse-Geisser correction indicated that the mean ratings of frequency were significantly different across the coping strategies (F(9.477,957.142) = 25.539, p < .001, η_p_^2^ = .20) and showed interactions between coping and child age groups (F(18.953,957.142) = 1.784,(p = .021, η_p_^2^ = .03).

**Fig 1:**
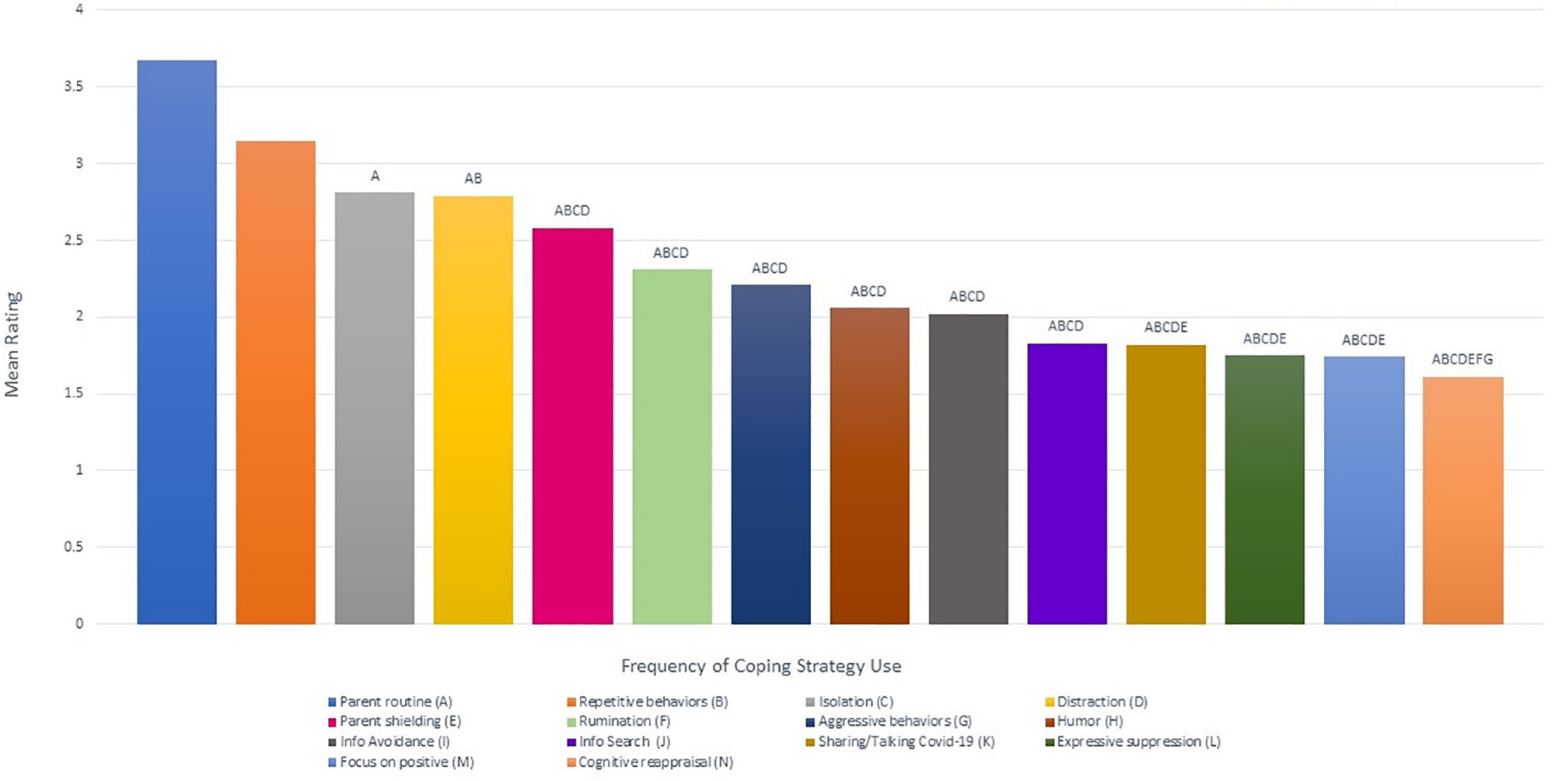
Pairwise comparisons between frequencies of coping strategies. The x-axis represents each of the 14 behavior survey questions. The letters represent all significant pairwise comparisons for the corresponding survey question.

We conducted post-hoc pairwise comparisons using Bonferroni corrections to compare the most and least frequent coping behaviors overall (Fig 1 and Table 3). Across the entire sample, ratings for *parent routine* were the most frequent strategy used and was significantly higher than all the other coping strategies (all p < .001) except for *repetitive behaviors* from which it did differ in frequency. Meanwhile *parent shielding* was used less often than parent routine (p < 0.001), and significantly more often than information search (p = 0.007), sharing/talking about COVID-19 (p = 0.002), focusing on the positive (p < 0.001), expressive suppression (p < 0.001) and cognitive reappraisal (p< .001).

Among maladaptive strategies, *repetitive behaviors* were the most frequently used (and the second most frequent strategy overall), with significantly higher ratings (all p < 0.001), than almost other strategies, except isolation, distraction, parent shielding, and parent routine. In addition, *isolation* was the third most frequently used coping strategy overall and was significantly higher than information search, sharing/talking about COVID-19, expressive suppression, focus on the positive and cognitive reappraisal (all p < 0.001), as well as information avoidance (p = 0.005) and humor (p = 0.012). Further, *rumination* and *aggressive behavior* were both used significantly less often than repetitive behaviors or parent routine (all p < 0.001), but significantly more often than cognitive reappraisal (p = 0.003 and p = 0.031 respectively).

With respect to adaptive strategies, *distraction* was used more often than information avoidance, information search, expressive suppression, focusing on the positive, and cognitive reappraisal (all p < 0.001), as well as sharing/talking about COVID-19 (p = 0.001) and humor (M = 2.06, SD = 1.23, p = 0.007). In contrast, *cognitive reappraisal* was the least frequently used strategy overall, and its ratings were significantly lower than parent routine, parent shielding, repetitive behaviors, isolation, and distraction (all p < 0.001) as well as rumination (p = 0.003), and aggressive behavior (p = 0.031).

Finally, *cognitive reappraisal*, *expressive suppression*, *focusing on the positive*, *sharing/talking about COVID-19*, *information search*, *information avoidance*, *and humor* did not differ significantly from one another in the frequency by which they were used as coping strategies.

We then examined post-hoc pairwise comparisons using Bonferroni corrections for the use of coping strategies across the various age groups (Table 5). Results revealed that the frequency of *isolation* was lower in the 3–9-year-old group (M = 2.13; SD = 1.26,) than either the 10–15-year-old group (M = 3.12; SD = 1.61, p = 0.019) or the 16–21-year-old group (M = 3.07; SD = 1.57, p = 0.050). In contrast, *parent routine* was more often established for children aged from 3 to 9 years old (M = 4.03; SD = 1.15) than for the 10–15-year-old group (M = 3.32; SD = 1.36, p = 0.044), whereas no significant difference was reported for children over 15 years old. Frequency of all other strategies did not differ between the different age groups.

**Table 5:** Frequency of coping strategies across age groups.

Coping strategies	Age group	Mean	*SD*
Adaptive strategies			
*Distraction*	3–9 years old	2.29	1.296
	10–15 years old	3.07	1.639
	16–21 years old	2.90	1.494
*Humor*	3–9 years old	2.19	1.250
	10–15 years old	2.12	1.199
	16–21 years old	1.83	1.262
*Sharing/Talking about COVID-19*	*3–9 years old*	1.87	.991
	10–15 years old	1.91	1.461
	16–21 years old	1.63	1.033
*Focusing on the positive*	3–9 years old	1.61	1.022
	10–15 years old	1.74	1.093
	16–21 years old	1.87	1.074
*Cognitive reappraisal*	3–9 years old	1.58	.807
	10–15 years old	1.53	.882
	16–21 years old	1.73	1.081

*Repetitive behaviors*	3–9 years old	2.90	1.578
	10–15 years old	3.42	1.500
	16–21 years old	3.03	1.564
*Aggressive behaviors*	*3–9 years old*	2.55	1.457
	10–15 years old	2.30	1.520
	*16–21 years old*	1.73	1.172
*Rumination*	3–9 years old	2.35	1.404
	10–15 years old	2.40	1.450
	16–21 years old	2.13	1.408
*Isolation*	3–9 years old	2.13	1.258
	10–15 years old	3.12	1.607
	16–21 years old	3.07	1.574
*Information Avoidance*	3–9 years old	1.87	1.147
	10–15 years old	1.95	1.379
	16–21 years old	2.27	1.574
*Expressive suppression*	3–9 years old	1.81	1.276
	10–15 years old	1.70	1.245
	16–21 years old	1.77	.935
*Information Search*	3–9 years old	1.74	1.094
	10–15 years old	1.88	1.434
	16–21 years old	1.83	1.177

*Parent routine*	3–9 years old	4.06	1.153
	10–15 years old	3.33	1.358
	16–21 years old	3.77	1.223
*Parent shielding*	3–9 years old	2.84	1.530
	10–15 years old	2.49	1.352
	16–21 years old	2.43	1.382

### Efficacy of coping strategies

Repeated measures ANOVAs with Greenhouse-Geisser correction indicated that the mean ratings of efficacy were significantly different across the coping strategies (F(9.856,995.474) = 15.233, p < 0.001, η_p_^2^ = 0.13) and there were interactions between strategies and child age groups (F(919.712,995.474) = 2.826, p < .001, η_p_^2^ = 0.05).

**Fig 2:**
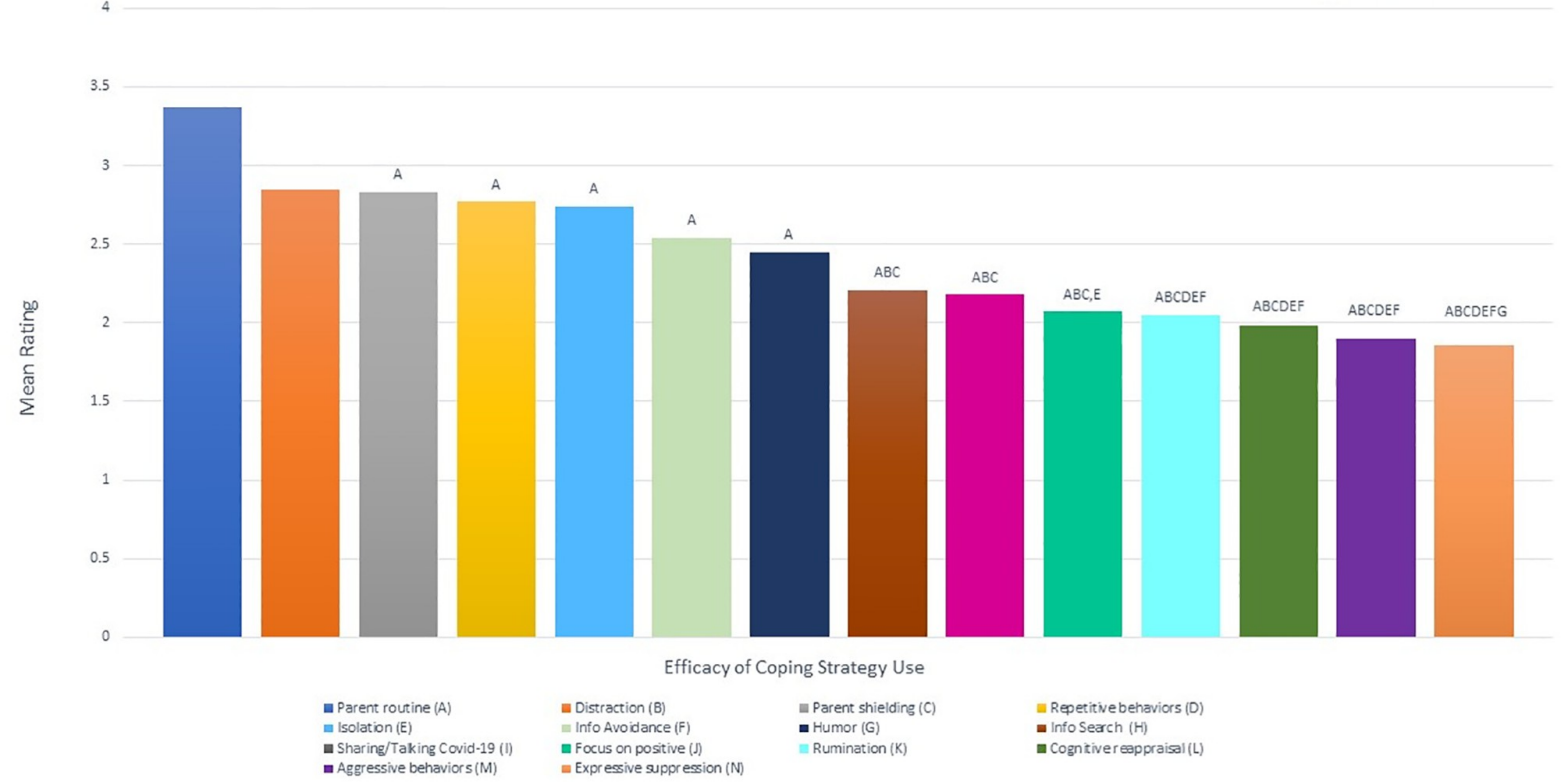
Pairwise comparisons between efficacies of coping strategies. The x-axis represents each of the 14 behavior survey questions. The letters represent all significant pairwise comparisons for the corresponding survey question.

Comparing the efficacy of coping strategies across the entire sample by pairwise comparisons using Bonferroni corrections (Fig 2, see Table 4), *parent routine* was rated as significantly more efficacious than almost of the other strategies (all at p < 0.001), as well as information avoidance (p = 0 .001), isolation (p = 0.009), repetitive behaviors (p = 0.012) and parent shielding (p = 0.043). Only *distraction* did not differ significantly in efficacy compared to *parent routine*. Meanwhile *parent shielding* was rated as the third most effective strategy overall and was significantly higher than rumination, cognitive reappraisal, aggressive behavior, and expressive suppression (all p < 0.001), as well as information search (p = 0.022), sharing/talking about COVID-19 (p = 0.008), and focusing on the positive (p = 0.004).

With respect to adaptive strategies, *distraction* was rated as the second most effective coping strategy, with higher scores than cognitive reappraisal, aggressive behavior, expressive suppression, and rumination (all p < 0.001), as well as focusing on the positive (p = 0.002), sharing/talking about COVID-19 (p = 0.027) and information search (p = 0.035). *Humor* was also more efficacious than expressive suppression (p = 0.002).

Among maladaptive strategies, *repetitive behavior* was rated as the most efficacious with significantly higher ratings than cognitive reappraisal (p = 0.022), rumination (p = 0.003), expressive suppression (p < 0.001) and aggressive behavior (p < 0.001). *Isolation* was also rated as more efficacious than cognitive reappraisal (p < 0.001), rumination (p < 0.001), expressive suppression (p < 0.001) and aggressive behavior (p < 0.001), as well as focusing on the positive (p = 0.003). *Information Avoidance* was rated as higher in efficacy than expressive suppression (p = 0.002), rumination (p = 0.015), cognitive reappraisal (p = 0.028), and aggressive behavior (p = 0.039).

Finally, *sharing/talking about COVID-19*, *focusing on the positive*, *cognitive reappraisal*, *information search*, *expressive suppression*, *rumination*, and *aggressive behavior* did not differ significantly from one another in their perceived efficacy.

Post-hoc pairwise comparisons using Bonferroni corrections were conducted to evaluate the efficacy of coping strategies across the different age groups (Table 6). Among maladaptive strategies, engaging in *aggressive behaviors* was rated as more efficacious in reducing stress for children ages 3 to 9 years old (M = 2.42; SD = 1.67) than for 16–21-year-olds (M = 1.53; SD = 1.17; p = 0.038), and neither group differed significantly from the 10–15-year-old group (M = 1.79, SD = 1.25). Regarding parents’ strategies, *parent shielding* was more efficacious for the younger children (3–9 years old: M = 3.48; SD = 1.41) than for the 16–21-year-olds (M = 2.27; SD = 1.39, p = 0.002) with no significant difference for either group from the 10–15 years old group (M = 2.74, SD = 1.33). There was no significant difference between age groups in efficacy for any of the other coping strategies.

**Table 6:** Efficacy of coping strategies across age groups.

Coping strategies	Age group	Mean	*SD*
Adaptive strategies			
*Distraction*	3–9 years old	2.71	1.442
	10–15 years old	2.84	1.542
	16–21 years old	3.00	1.083
*Humor*	3–9 years old	2.65	1.539
	10–15 years old	2.25	1.386
	16–21 years old	2.50	1.570
*Sharing/Talking about COVID-19*	*3–9 years old*	2.52	1.435
	10–15 years old	2.07	1.486
	16–21 years old	2.00	1.259
*Focusing on the positive*	3–9 years old	2.10	1.446
	10–15 years old	1.77	1.192
	16–21 years old	2.47	1.408
*Cognitive reappraisal*	3–9 years old	2.03	1.239
	10–15 years old	1.70	1.103
	16–21 years old	2.33	1.373

*Information Search*	3–9 years old	2.61	1.564
	10–15 years old	2.07	1.404
	16–21 years old	2.00	1.287
*Repetitive behaviors*	3–9 years old	2.55	1.588
	10–15 years old	3.14	1.355
	16–21 years old	2.47	1.252
*Information Avoidance*	3–9 years old	2.45	1.480
	10–15 years old	2.26	1.449
	16–21 years old	3.03	1.402
*Aggressive behaviors*	*3–9 years old*	2.42	1.669
	10–15 years old	1.79	1.245
	*16–21 years old*	1.53	1.167
*Isolation*	3–9 years old	2.39	1.498
	10–15 years old	2.81	1.484
	16–21 years old	3.00	1.141
*Rumination*	3–9 years old	2.10	1.326
	10–15 years old	2.05	1.154
	16–21 years old	2.00	1.203
*Expressive suppression*	3–9 years old	1.65	.950
	10–15 years old	1.79	1.283
	16–21 years old	2.17	1.262

*Parent routine*	3–9 years old	3.55	1.234
	10–15 years old	3.00	1.363
	16–21 years old	3.70	1.317
*Parent shielding*	3–9 years old	3.48	1.411
	10–15 years old	2.74	1.329
	16–21 years old	2.27	1.311

### Correlations between frequency and efficacy of coping strategies

Overall, Spearman’s rank order correlation analyses indicated significant correlations between frequency and efficacy all but two of the coping strategies as described in Table 7.

**Table 7:** Spearman’s rank order correlation coefficients for coping strategies.

Coping strategies	Age group	Correlation coefficient *rs*	*p value*
Adaptive strategies			
*Cognitive reappraisal*	3–9 years old	**.667**	< 0.001
	10–15 years old	**.693**	< 0.001
	16–21 years old	**.539**	0.002
*Focusing on the positive*	3–9 years old	**.657**	< 0.001
	10–15 years old	**.965**	< 0.001
	16–21 years old	**.547**	0.001
*Humor*	3–9 years old	**.808**	< 0.001
	10–15 years old	**.792**	< 0.001
	16–21 years old	**.649**	< 0.001
*Sharing/Talking about COVID-19*	3–9 years old	.339	0.058
	10–15 years old	**.393**	0.008
	16–21 years old	**.399**	0.026
*Distraction*	3–9 years old	**.550**	0.001
	10–15 years old	**.714**	< 0.001
	16–21 years old	**.598**	< 0.001
**Maladaptive strategies**			
*Information Search*	3–9 years old	**.484**	0.004
	10–15 years old	**.496**	< 0.001
	16–21 years old	**.490**	0.005
*Information Avoidance*	3–9 years old	**.596**	< 0.001
	10–15 years old	**.617**	< 0.001
	16–21 years old	**.599**	< 0.001
*Expressive suppression*	3–9 years old	**.612**	< 0.001
	10–15 years old	**.474**	< 0.001
	16–21 years old	**.453**	0.011
*Rumination*	3–9 years old	**.451**	0.010
	10–15 years old	**.639**	< 0.001
	16–21 years old	**.498**	0.004
*Isolation*	3–9 years old	**.511**	0.003
	10–15 years old	**.635**	< 0.001
	16–21 years old	**.658**	< 0.001
*Aggressive behaviors*	3–9 years old	.190	0.299
	10–15 years old	**.391**	0.009
	16–21 years old	.355	0.055
*Repetitive behaviors*	3–9 years old	**.717**	< 0.001
	10–15 years old	**.425**	0.003
	16–21 years old	**.416**	0.020
**Parents’ strategies**			
*Parent shielding*	3–9 years old	**.559**	< 0.001
	10–15 years old	**.651**	< 0.001
	16–21 years old	**.727**	< 0.001
*Parent routine*	3–9 years old	**.510**	0.003
	10–15 years old	**.663**	< 0.001
	16–21 years old	**.537**	0.002

### Children’s coping strategies

The frequency and efficacy ratings were associated for nearly all coping strategies across all age groups (Table 7). However, there were some differences according to age. With respect to children’s adaptive coping strategies, there were significant correlations for all strategies with the exception of except *sharing/talking about COVID-19* for the 3–9-year-old group, for whom the frequency of was not correlated to its efficacy on reducing stress. For the youngest (3–9 years old) and the oldest (16–21 years old) children, frequency of *humor* was the most correlated with its efficacy (rs = 0.81, p < 0.001 and rs = .65, p < 0.001, respectively), whereas *focusing on the positive* had the highest correlation coefficient between frequency and efficacy for children between 10 and 15 years old (rs = .97; p < 0.001) (Tables 5 and 6). It should be noted, however, that neither of these two strategies were rated among the most frequently used nor the most effective coping strategies within any age group (Tables 5, 6 and Fig 2).

Strong correlations were also found among all of the maladaptive strategies (Table 7). All correlations were significant except for *aggressive behaviors* for the youngest group (3–9 years old) and the oldest group (16–21 years old). Depending on the age group, the highest correlation coefficients were: *repetitive behaviors* for the 3–9-year-old group (rs = 0.72; p < 0.001), which was also rated the most frequently used and the most efficient maladaptive strategy within this group; *rumination* for children 10–15 years old (rs = .64; p < 0.001); and *isolation* for those from 16 to 21 years old (rs = 0.66; p *<* 0.001), which had the highest ratings of frequency and efficacy for this group (Tables 5 and 6).

Interestingly, significant correlations between frequency and efficacy for two paradoxical strategies were found for children from all age groups: getting as much information as possible about COVID-19 (*information search*) and avoiding information about the situation (*information avoidance)* (Table 7).

### Parents’ strategies

Frequencies of parents’ strategies (*parent shielding* and *parent routine*) were closely related to their efficacy in reducing stress, regardless of their child’s age (Table 7).

## Discussion

A common feature of children with ASD is a strict follow up with their schedule, and disruption of their routines can cause them significant distress [46]. Due to this, individuals with ASD are particularly impacted by the COVID-19 pandemic [10, 47, 48]. Adapting to new daily routines, environmental changes, and uncertainty related to the pandemic have been determined to be causing distress in children with ASD, worsening both the core features of ASD and their overall mental health [49, 50]. To manage distress linked to COVID-19, children with ASD were shown in one study to be using a disengagement coping response [51].

This study aimed to analyze the frequency and efficacy of several different coping response strategies, including both adaptive and maladaptive strategies, used by school-aged children with ASD and their parents during the COVID-19 pandemic. It is hoped that this work will contribute to a growing body of literature exploring the specific coping and emotional regulation strategies used by children with ASD. Although this study examined coping strategies during the pandemic, our finding may have implications for stress management in contexts other than future public health crises. In addition, we also explored age related differences in the use and effectiveness of specific coping strategies among children with ASD. To our knowledge, this is an area that has been explored in only a few studies of individuals with ASD to date [22, 30, 45]. Finally, we conducted exploratory correlational analyses to evaluate the extent to which ratings of frequency and perceived efficacy for each coping strategies are related, an area which has not yet been studied in individuals with ASD.

### Overall frequency and efficacy ratings

With respect to the frequency and efficacy of parent ratings of the different child coping strategies measured in the current study, it is important to note that ratings of frequency and efficacy were low for nearly all strategies in the survey, in that they rarely exceeded values of “3” (moderately frequent and effective). This finding suggests that, overall, the majority of strategies were used infrequently and not viewed as particularly effective in managing stress even when used.

Comparing strategies to one another, variances were noted with respect to the frequency and efficacy. Overall, results indicate that children in this sample used *maladaptive* strategies more frequently than *adaptive* strategies. Indeed, of the five most frequently utilized child coping strategies, four are considered to be maladaptive, namely *repetitive behaviors*, *isolation*, *rumination* and a*ggressive behaviors*. This is consistent with previous research indicating that children with ASD are more likely to use maladaptive coping and emotional regulation strategies [22, 23, 30]. Further, these same four maladaptive strategies were also rated among the top five strategies with respect to efficacy, which a novel finding. This finding is consistent with the idea that maladaptive behaviors may be used by individuals with ASD to cope with anxiety and stress [31–39], and is also consistent to research with other populations showing that maladaptive strategies (such as catastrophizing and alcohol use) can be rated as effective stress-reducing strategies [41, 52]. Given the association between maladaptive strategy use and psychological distress and poor mental health [18, 22, 53], efforts should be made to assist individuals with ASD to develop more adaptive strategies as replacements.

With respect to *adaptive strategies*, children with ASD in our sample rarely used *focusing on the positive* or *cognitive reappraisal*, and the rated efficacy of these strategies was also low. While focusing on the positive has received less attention in the ASD literature, research has documented that individuals with ASD use cognitive reappraisal less frequently and with less efficacy [23, 30, 54]. Additionally, it is important to note that while adaptive strategies were utilized less often overall, *distraction* was used with a higher degree of frequency than other adaptive strategies in our sample and was also rated as the most efficacious of all the child coping strategies. This is consistent with the findings from Ma and colleagues [44] who found that informal coping strategies employed by parents, such as diversion (i.e., distraction), were associated with decreased problem behavior in their children. Furthermore, although it was used with less frequency among our sample, parents rated *humor* as a coping strategy higher in effectiveness than all other adaptive strategies other than *distraction*. These findings suggest that interventions focused on helping children with ASD cope with stress may benefit from including strategies such as humor and distraction among their skill development repertoire.

However, results suggest that interventions aimed at helping individuals change how they are thinking or viewing the situation may be less successful. In part, this may be due to the executive functioning difficulties and inflexible thinking patterns common among individuals with ASD, which may impact their ability to use more cognitive coping strategies [32, 52]. The finding that individuals with ASD with and without intellectual disability did not differ in their use cognitive appraisal lends support for this idea [52]. Nonetheless, there is some evidence that children with ASD can be prompted to engage in cognitive appraisal and benefited from this strategy when used [23], indicating the need for further research to better understand how to intervene in this area and determine who is most likely to benefit from such interventions.

Finally, parent-mediated coping strategies, such as *parent routine* and *parent shielding* were rated among the most frequently used and efficacious within our sample. In fact, *parent routine* was the most highly rated for both frequency and efficacy. Given the strong reliance on routines among children with ASD, coupled with the significant disruption to normal schedules and daily activities during the pandemic, it is not surprising that parents would take an active role in helping their child manage this stress. Our finding is consistent with previous research that suggests the usefulness of parenting strategies during the pandemic, such as offering behavioral and language comfort, diverting their child’s attention, and creating a routine that offers access to activities such as play, music and exercise [44, 55].

### Age-related differences in frequency and efficacy ratings

There were some significant differences between age groups in the frequency ratings of different strategies. For example, *isolation* was used less often by children from 3 to 9 years of age in comparison to both other age groups, while *parent routine* was used more frequently with children from 3 to 9 years old when compared to the oldest age group. This dissimilarity is likely due to the increased independence and greater behavioral autonomy of older children and adolescents, as compared to younger children who rely more on their parents for their overall well-being and emotion regulation [25, 28].

In terms of efficacy, no significant age differences in the efficacy of any of the adaptive strategies was seen. *Aggressive* behaviors and *parent shielding* however were both rated as more effective for the youngest age group. The finding that *parent shielding* was rated as more effective by parents of younger that older children is not surprising since younger children generally rely more on their parents to help them cope with stressful situations and regulate emotions and may be more vulnerable to negative outcomes due to their less well-developed coping skills [28. 32]. The finding that *aggression* was rated as an effective stress-management behavior is consistent with previous research highlighting that aggression may serve an escape function which helps the child disengage from unwanted interactions or demands [37–39]. If this is the case, it suggests that interventions targeting replacement coping strategies that serve the same function might be effective in helping develop more adaptive coping responses. However, the finding that by parents of the youngest children rated this behavior as more effective than parents of older children bears further study. It may be that the current findings simply reflect the tendency toward higher levels of aggression in younger children with ASD, which have been noted in several studies [56, 57]. This may suggest that interventions aimed at teaching replacement coping skills may be especially important for families of younger children.

### Correlations between frequency and efficacy ratings

We observed significant positive correlations between the frequency and efficacy ratings for nearly all coping strategies across all age groups. In other words, the more frequently a strategy was used, the more effective it was rated; conversely the less frequently it was used, the less effective it was rated. This was true for both adaptive and maladaptive strategies. With respect to adaptive strategies, it makes intuitive sense that adaptive strategies would be seen as more efficacious for children with ASD when they are used with higher frequency. Interpreting the positive correlations between the frequency of maladaptive strategy use and their efficacy is less straight-forward, but is also consistent with previous research [41, 52]. Given the low frequency of many of the coping strategies, positive correlations could reflect the “infrequent use of ineffective strategies” [41]. Conversely, finding could also indicate that maladaptive coping strategies are in fact effective when used with frequency. For example, catastrophizing was found to be rated as an effective strategy in managing pain for those who frequently used that strategy in one study [41], while alcohol use was rated as effective in reducing stress among mental health providers during the pandemic in another [52].

In this respect, it is important to note that the direction of the association between frequency and efficacy ratings cannot be determined from the present study. It may be that strategies which are used more frequently become more effective through practice. Rather, it may be that strategies that are more effective in reducing stress, whether adaptive or maladaptive, come to be used more frequently through reinforcement [32]. Further research into the directionality of this association is needed. In particular, longitudinal studies that examine coping strategy development over time may assist us in fully understanding these results and their implications for intervention.

Results further revealed strong correlations between efficacy and frequency of parent-mediated strategies (*parent routine* and *parent shielding)* for all three age groups. Stay-at-home orders during the pandemic necessitated a more active parenting role in their child’s education, and a need to balance working at home with other child giving demands [9, 10]. Further, news regarding the pandemic’s effects is likely to have impacted parenting stress and anxiety, as well as for their child [13, 17, 18]. Thus, it is not surprising to find strong positive correlations between parent’s ratings of the frequency and efficacy of attempts at managing their child’s stress.

Finally, results revealed some *age-related differences* in correlation patterns for both adaptive and maladaptive coping strategies. Strong positive correlations between frequency and efficacy were found for the adaptive coping strategies of *humor* and *focusing on the positive* across all age groups. However, as noted above, these strategies were not utilized with a high level of frequency overall. The finding that, when used, they are highly effective, suggests that teaching these types of adaptive behaviors can be advantageous for children with ASD in managing their stress and regulating their emotions regardless of age. This is consistent with results from a previous study which revealed that parents of children with ASD from ages 3 to 8 frequently reported their child’s sense of humor as a social personality strength [58]. Interestingly the lowest correlations were found for *sharing/talking about COVID-19* suggesting that even when used, it is not likely to be particularly effective, which is consistent with literature suggesting that this strategy might be linked to higher levels of anxiety during the pandemic [59]. This information can be useful in determining which adaptive coping and emotion regulation strategies to encourage amongst children of different age groups in order to provide the greatest relief.

Maladaptive coping strategies were also variable in their patterns of correlation across all age groups. For children between 3 and 9 years old, *repetitive behaviors* had the highest correlation between frequency and efficacy; this may be because these young children have received less intervention, such as ABA therapy, and therefore have not yet developed more effective alternative coping and emotion regulation strategies. Meanwhile, *rumination* had the highest reported correlations for 10- to 15-year-old while, *isolation* had the strongest correlation amongst the 16 to 21 age group. It does make sense that children in the 10 to 15 age group would be more likely to utilize cognitive coping strategies, such as *rumination*, as these tend to develop in middle childhood to later childhood [25]. However, it is unclear why there would be such a strong correlation between its frequency and efficacy. Rumination involves obsessive and intrusive thoughts about a particular topic and has been linked to an increased risk of psychopathology [25]. It may be that this particular question was poorly understood by respondents since the term rumination was not defined on the questionnaire. Thus, replication of this finding is needed to fully understand the role of rumination as an effective coping strategy for children with ASD. With respect to *isolation*, while this is considered a maladaptive strategy for the purpose of this study, the significant positive correlation between its frequency and efficacy suggest it may be adaptive given the context of the pandemic. Specifically, research has documented increases in conflict among families of children with ASD during the pandemic due to stay-at-home policies [12, 13]. Isolation may be an efficacious way of diffusing tension for older children who are more able to successfully utilize this strategy.

The literature has revealed that “a high emotional climate,” similar in nature to that of the COVID-19 crisis, is correlated with an elevated occurrence of maladaptive behavior in ASD over time [30]. This may be attributed to the fact that increased and intensified negative behaviors are twice as likely to occur in individuals who presented with behavior difficulties prior to the pandemic [17]. Repetitive behaviors, mannerisms, stereotypes, and sleep regulation problems were found to be higher in children with ASD as a result of the pandemic [60, 61]. Moreover, quarantine during COVID-19 has been found to have a negative impact on emotion management and a higher level of anxiety in ASD children than in typically developing children [62]. Because this survey did not inquire about pre-COVID-19 problem behaviors or ASD severity, it is possible that many of the children whose parents participated in this study had a previous history of less severe behavioral outbursts and/or autism traits.

In the literature, there are many studies about coping mechanisms of parents of children with ASD, both with and without respect to the COVID-19 pandemic [63–67]. But there is relatively less literature which focuses on the way in which how children with ASD cope with the stress created by the COVID-19 pandemic. It is important to recognize the needs of these children. particularly with regard to the impact of the pandemic on mental health and post-pandemic mental disorders. Findings from the current study provide some insight into the specific coping strategies used by children with ASD, the perceived efficacy of those strategies, and how these may differ across age groups. Future research should explore whether level of autism severity or co-occurring intellectual disability impacts coping strategy use and effectiveness. In addition, it will be important to explore the frequency and perceived efficacy of various coping strategies across a variety of contexts and specific stressors. Such results will have implication beyond future public health emergencies, as they may allow us to develop interventions designed to teaching effective and positive coping strategies to parents and children, and potentially vary strategies according to children’s unique symptom presentation as well as the context in which they are most likely to be effective.

### Limitations

The results of this study provide vital information regarding the adaptive and maladaptive coping strategies that children with ASD in the US are using during the COVID-19 pandemic. One of the limitations of this study is that it utilizes a relatively small cohort of children with ASD in the US. Therefore, it may not be representative of the lived experiences of all individuals with ASD who are using these coping strategies to alleviate stress, and it is unclear whether these findings would replicate to other locations or for other stressors beyond the pandemic.

Additionally, this study involved the use of parent ratings of the frequency and efficacy of the various coping strategies used by children, rather than the children providing these ratings for themselves; this makes it difficult to determine the validity of the parent ratings in this study. While research indicates that there is generally only moderate agreement between pare-report and child self-report [68, 69], there may be greater overlap between parents’ reports and their children’s self-reports on coping questionnaires [24]. Nevertheless, it is likely that parents may not be able to accurately identify the frequency of more internally mediated strategies such as cognitive reappraisal, rumination, expressive or emotional suppression, and focusing on the positive as these may not be as observable. Further, although parents may be able to evaluate the effectiveness of a coping strategy based on alterations in the child’s behavior and mood, it may be more difficult for the parent to truly know whether the specific strategy has resulted in a decrease in internalized stress and anxiety for their child. Due to the lockdown, it was not possible to confirm the validity of parents report by professionals, suggesting a need to develop tools and scales to validate parent responses especially during public health emergencies such as pandemics.

An important and related limitation is that validity of parent report may be especially compromised for children with more moderate to severe intellectual disability, who may not be able to proactively adopt more cognitively mediated strategies (such as changing the way he or she is thinking about the situation or focusing on positive aspects) which may require higher levels of cognition. However, our sample was selected to only include participants whose parents indicated they were aware of the COVID-19 pandemic) resulting in the majority of those with intellectual disability in our sample falling in the mild/moderate versus severe range. These participants have more situational understanding of their surroundings, and therefore may be more able to utilize cognitive coping strategies. We are further encouraged by the findings of a recent publication which compared the coping strategy use among children with ASD with and without intellectual disability [45]. While this study revealed a significant difference between the two groups in *focusing on the positive*, neither group utilized this strategy very frequently. Further, no group differences were found in the use of *cognitive reappraisal*. These findings suggest that cognitive coping strategies are used infrequently by individuals with ASD regardless of level of intellectual functioning, which give some confidence to our findings. However, it is important to note that this study did not report the distribution of mild/moderate to severe intellectual disability. Therefore, it will be critically important for future research to explore the impact of level of co-occurring ID (mild, moderate, severe) among individuals with ASD on the frequency and effectiveness of coping strategy use.

In addition to the level of intellectual disability, medication use and other health issues may all interact to impact the frequency of specific emotion regulation and coping strategy use, as well as on the effectiveness of such strategies. These were not examined in the current study, but are important to address in the future studies, as they may represent influencing factors in the emotions and coping styles of children with ASD.

Finally, another limitation of the design of our questionnaire is that the frequency and efficacy ratings were made separately for each strategy, while in practice, parents and children likely employ more than one strategy at the same time. Future studies are warranted to determining which combinations of coping mechanisms are more effective in mitigating stress in children with ASD, especially considering that the use of multiple strategies has been linked to lower levels of problem behavior in children with intellectual disability during the pandemic [44].

### Implications

Due to the perceived differences in coping and emotion regulation strategy use and efficacy demonstrated through this study, implementation of a “strength-based approach” to ASD evaluation, and coping and emotion regulation development, could be beneficial for children with ASD [28, 31]. Whereas there is more available literature relating to their parents’ and families’ emotion regulation and stress-reduction techniques, limited research focus has been placed on the emotion regulation and coping abilities of children with ASD. This study aimed to expand the knowledge base in this area.

Although our results suggest that maladaptive coping strategies can be effective, we would suggest that parents and therapist focus on assisting children to develop more adaptive strategies. Individuals with ASD have higher anxiety levels and also commonly experience emotion dysregulation which has been associated with maladaptive behaviors [30, 62, 70, 71]. Additionally, decreased coping abilities are associated with increased severity of ASD, indicating the importance of teaching children with ASD effective coping and emotion regulation strategies in order to reduce the occurrence of negative behaviors [31]. However, it is important to keep in mind that the abrupt termination of the maladaptive coping or emotion regulation strategies that the child finds comforting may result in increased stress levels and disruptive behaviors. As a result, it may be more disruptive to cease the child’s use of these techniques prior to providing a more suitable replacement method for stress reduction.

Our hope is that this review of coping strategy use in the early months of the pandemic will serve as a useful guide to therapists and parents for promoting similar adaptive ER strategies when necessary. Further studies involving a larger cohort are warranted to more accurately determine effective ER strategies for individuals with ASD, especially for future public health emergencies. In addition, future studies should consider the role of parent stress/coping/mental health on their ratings of the child’s stress and coping. It will be worthwhile to see how the parents’ own experiences during the COVID19 pandemic and their perception of how well they managed their own stress was related, if at all, to their perceptions of the child’s stress and coping. Future research should also aim to include direct assessment of the experiences of individuals with ASD themselves across all levels of intellectual functioning.
